# Supplementary material for: Genome-wide mutational biases fuel transcriptional diversity in the Mycobacterium tuberculosis complex
Source: Nat Commun. 2019 Sep 5;10:3994. doi: 10.1038/s41467-019-11948-6 (PMC6728331; doi:10.1038/s41467-019-11948-6)
Supplement: Supplementary file 1 — Supplementary Information [file 41467_2019_11948_MOESM1_ESM.pdf]

Genome-wide mutational biases fuel transcriptional diversity in the *Mycobacterium tuberculosis* complex

Álvaro Chiner-Oms[1#], Michael Berney[2#], Christine Boinett[3,4], Fernando González-Candelas[1,5], Douglas Young[6], Sebastien Gagneux[7,8], William R. Jacobs Jr[2], Julian Parkhill[3], Teresa Cortes[9]*, Iñaki Comas[5,10]*.

1- Unidad Mixta "Infección y Salud Pública" FISABIO-CSISP/Universidad de Valencia, Instituto de Biología Integrativa de Sistemas-I2SysBio, Valencia, Spain.
2- Albert Einstein College of Medicine, Department of Microbiology and Immunology, New York, USA.
3- Sanger Institute, Wellcome Genome Campus, Hinxton, Cambridge, UK
4- Hospital for Tropical Diseases, Wellcome Trust Major Overseas Programme, Oxford University Clinical Research Unit, Ho Chi Minh City, Vietnam
5- CIBER en Epidemiología y Salud Pública, Valencia, Spain
6- The Francis Crick Institute, London, UK.
7- Swiss Tropical and Public Health Institute, Basel, Switzerland.
8- University of Basel, Basel, Switzerland.
9- Infection Biology Department, Faculty of Infectious and Tropical Diseases, London School of Hygiene and Tropical Medicine, London, UK.
10- Instituto de Biomedicina de Valencia, IBV-CSIC, Valencia, Spain.

[#] These authors contributed equally
* Corresponding author

Supplementary Notes

A closer look into the PDEG genes

The two main groups in the MTBC phylogeny are MAF and MTB. As stated in the main text, the short genetic distance between both groups contrasts with a high number of differentially expressed genes. 18 genes were significantly upregulated and 9 were significantly downregulated between both groups (BH adjusted p-value < 0.05, fold-change >1.5). Despite the *mbt* and *ctp* genes, there are other interesting genes that showed differential expression between MAF and MTB. Gene Rv0216, which is known to be essential for bacterial survival during infection, is overexpressed in MAF[1]. It is also interesting that ncRNA-mcr16, a non-coding RNA located in the *fadB* locus, appears as differentially down-regulated in MTB in comparison with MAF. FadB is involved in mycolic acid synthesis[2] and the PDEG of this ncRNA could be potentially involved in transcriptional regulation of FadB.

Regarding the PDEG genes between L5 and L6, some of the highest differences in expression are found in the toxin-antitoxin systems VApBC3 and VapBC5, which are upregulated in L6. Toxin-antitoxin systems have been proposed to play a role in response to stress. Specifically, VapBC3 and VapBC5 are up-regulated in moderately low pH conditions (i.e., the phagosome)[3]. As the pathogen is able to reside in the acidic phagosome during infection[4], the basal up-regulation of this system in L6 could be related to adaptation to the host environment during disease progression. Also the genes related to the copper ion response are upregulated in L6. Copper ions affect bacteria during the infectious process and the management of high levels of this substrate is required for full virulence in animal infections[5]. With respect to L5, the most upregulated gene is *acyP,* a gene that encodes an acylphosphatase involved in the pathway of pyruvate metabolism[6]. Also *nirB* and *nirD*, which are known to play a role during dormancy[7] were upregulated.

We have pointed out that one of the most upregulated genes in L1 is *virS*, a gene that regulates the *mymA* operon activity in specific conditions [8]. The *mymA* operon is known to be required for growth in macrophages and spleen. The observed upregulation of *virS* however, seems to have no effect on *mymA* regulation in this condition, as previously reported[9]. It is also interesting that *mpt63*, which encodes an epitope recognized by the immune system[10], had a strong antisense signal in lineage 1 strains.

The modern lineages form a monophyletic clade which is ~300 SNPs distant from the common ancestor of all the MTB strains. In this branch only 8 genes are upregulated. From these genes, 3 of them seem to form an operon (*nrdE*, *nrdI* and *nrdH*). NrdE is an essential protein involved in DNA replication and its transcriptional levels vary according to oxygen level[11,12]. Interestingly, these genes are significantly down-regulated in the lineage 1 strains.

Some of the few downregulated genes in L4 form part of the *mce2* operon. *Mce2* mutant strains showed an attenuated phenotype in the mouse model of infection, with less pro-inflammatory cytokine recruitment and less mortality rates in comparison with the wild-type[13].

Natural selection shapes sigma factor's recognition motifs abundance

With the aim of testing the effect of selection over σ factor's recognition motifs, we have performed a permutation test (Fig 6b). Briefly, we have simulated the effect of random mutations by introducing in arbitrary genomic positions the 8,093 nucleotide changes identified in the 19 MTBC strains, and this process has been repeated 1,000 times. By doing this we obtained, for each σ factor, a distribution of the expected new/disrupted motifs due to stochastic processes.

For each σ factor, a different picture emerges (Supplementary Figure 3). As stated in the main text, the appearance of new SigA recognition motifs seems to be maintained by the action of positive selection. Contrarily, negative selection seems to be acting to conserve the number of SigE and SigG recognition motifs. The number of new and disrupted motifs for SigE (z-scores of -7.80 and -6.93) and SigG (z-scores of -4.39 and -5.67) are less than expected by chance, thus pointing to the action of negative selection in these cases. Finally, for SigJ the number of observed events fall within the distribution of values expected by chance, suggesting that the selection is not acting to maintain the number of recognition motifs for this σ factor (Supplementary Figure 3).

New Pribnow boxes increases the transcription of nearby genes

A mutation previously reported in L3 upstream of the *ahpC* gene creates a new TANNNT motif in both the forward and reverse strands. This new box could be the cause of the observed overexpression of *oxyR*, *ahpC* and *ahpD* in all L3 strains reported in the section above (Supplementary Data 4 and Supplementary Data 5). We have found two other variants in the *oxyR*-*ahpC* intergenic regions, G2726051A and C2726121T. None of them are present in L3 strains and are not involved in the creation of new TANNNT motifs. G2726051A is present in all the L1 strains, and we did not see an increase in *ahpC* expression levels in this clade. C2726121T is only found in the N1177 strain (L6), which is the one that we have omitted from our analyses as it harbours the *rpoB* mutation.

Another interesting case is the upregulation of the ribonucleotide reductase genes *nrdE*, *nrdI* and *nrdH* (Rv3051c-Rv3053c operon) in the modern lineages (Supplementary Data 4). The

expression of these genes are regulated by the transcription factor NrdR[14]. It was previously hypothesized[15] that a new TSS created by the G3415332A variant present in all the modern lineages could potentially lead to NrdR-independent expression of the operon. Our results support this prediction, as we observe an upregulation of these genes in the strains that harbour this mutation. One more obvious case was the previously reported G3500149A SNP present in Beijing strains which leads to the overexpression of the *dosR* regulon[9].

Independently of the genetic context in which the Pribnow box appears there is always an increase in transcription rates of nearby genes. However, new boxes enhancing the transcription of complete genes or operons (i.e. those falling in intergenic regions) are more probable to be functionally relevant. In our analysis, we detected that the intergenic region located upstream the *narG* gene accumulates 2 variants that create 4 different TANNNT motifs (Supplementary Data 5). The C1287112T variant is found in the common branch of the modern lineages and seem to upregulate the transcription of the *narG* in the modern lineages compared with L6 and and L1 (Supplementary Figures 4a, 4b and 4d). NarG is a nitrate reductase known to be related with the switch from dormancy to active state[16]. L5 had also the NarG operon upregulated, although we had not found any new Pribnow box upstream *narG*. The fact that L5 had *nirB* and *nirD* upregulated too (Supplementary Data 5) suggested the involvement of a global regulator of the nitrogen metabolism. In concordance to that, we detected that Rv0260c is upregulated. In a past publication, we shown that the genes regulated by Rv0260c were those related with nitrate assimilation[17] so the overexpression of Rv0260c could be responsible for the upregulation of the genes involved in nitrate metabolism. The other variant found was C1287068T, which creates a new TANNNT motif in N0153 (L1) and increases the expression of the operon in N0153 with respect to the other L1 strains (Supplementary Figure 4C). The circumstance that L5 along with the modern lineages and some L1 strains had *narG* upregulated, cause that in our PDEG analysis the *narG* operon appear as downregulated in the L6 branch (Supplementary Data 4). To gain a global understanding of the abundance of these variants, we checked their distribution in a larger dataset (n=4,595)[18] containing clinical samples representative of the global MTBC diversity. In addition to the variants reported above, we found 2 more variants, C1287081T and G1287182T, that also created a new Pribnow box. Interestingly, all the variants that generate new Pribnow boxes upstream were found to be highly homoplastic (Supplementary Figure 4a) suggesting the action of positive selection. Most of our analyses can only be focused in the deeper branches of the phylogeny. However, the data from *narG* suggests that new Pribnow boxes fuelled by genome-wide mutational biases is a mechanism under selection to access transcriptional diversity in clinical strains.

Supplementary Figures

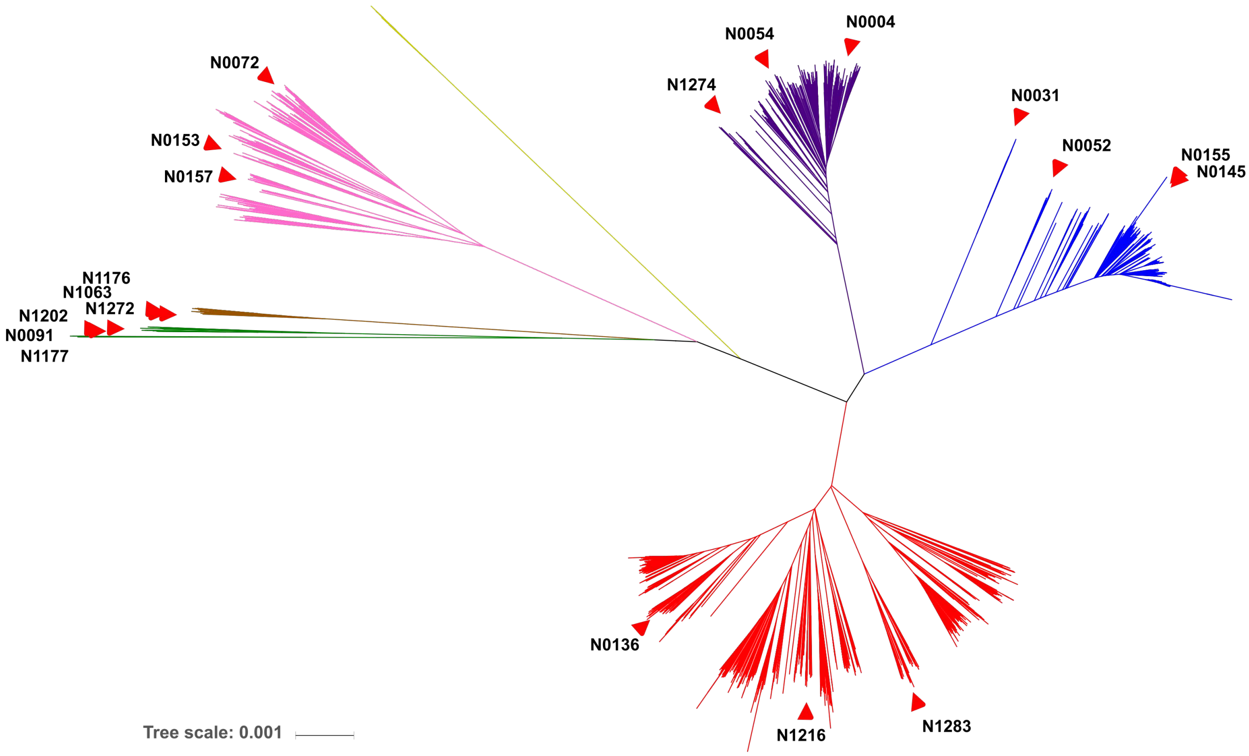

Supplementary Figure 1. Maximum-likelihood phylogeny, constructed with 4,595 strains representative of the global MTBC diversity[18]. Red marks point to the 19 strains used for the main analyses.

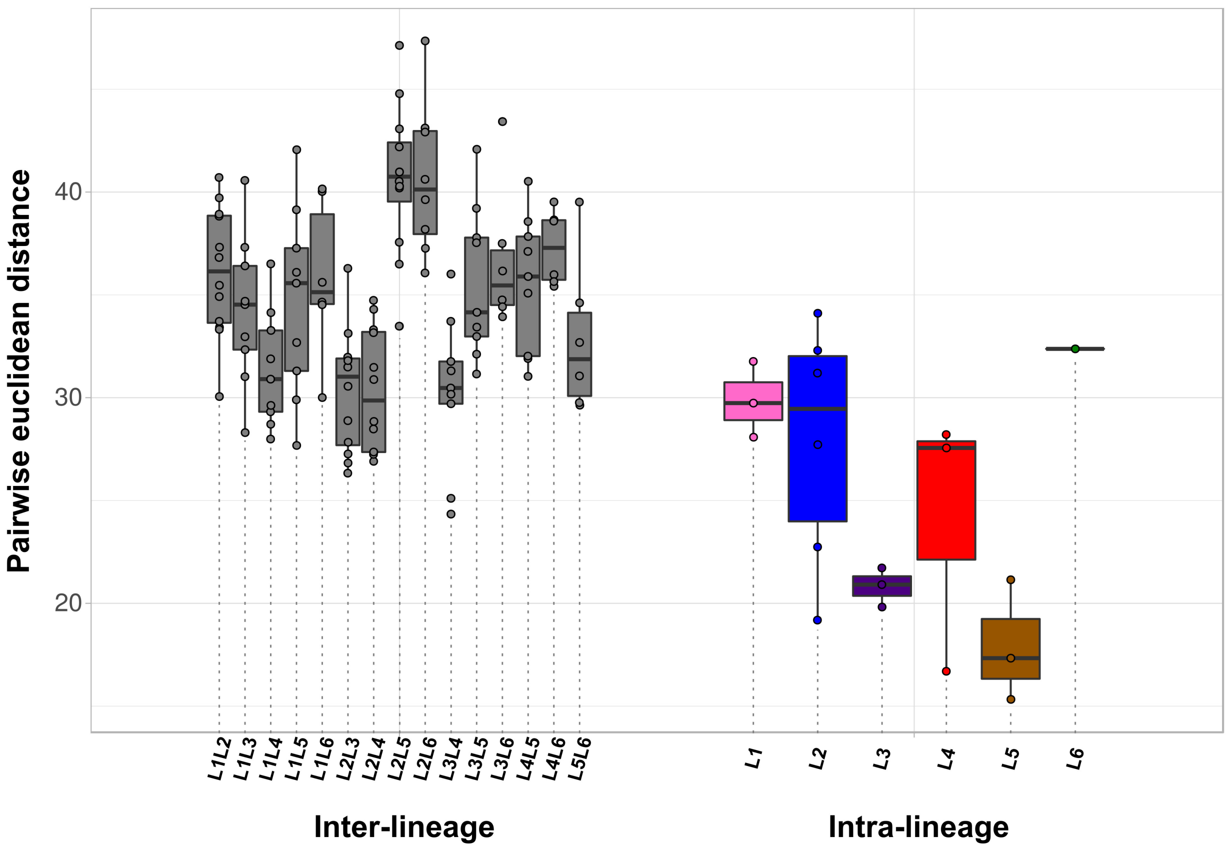

Supplementary Figure 2. Intra- and inter-lineage pairwise euclidean distance distribution, calculated from the complete transcriptomic data. This pattern of variability resembles the within-lineage pairwise SNP distance pattern[19].

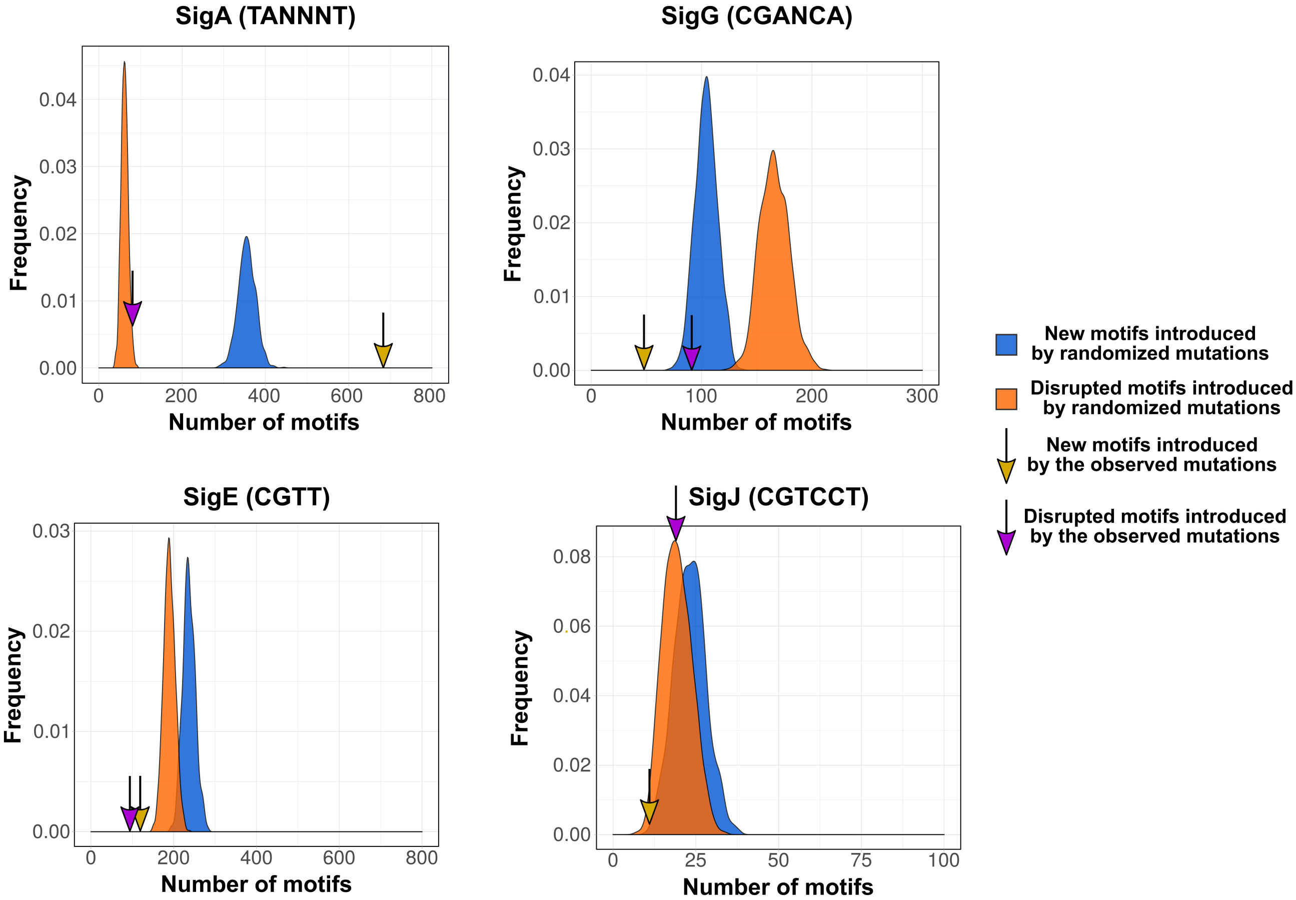

Supplementary Figure 3. Heterogeneous impact of the observed mutations over the sigma factors recognition motifs. New SigA recognition motifs are introduced in the MTBC genome at a higher rate than expected by chance, pointing to the effect of non-stochastic processes in the accumulation of new TANNNT motifs. In contrast, SigG and SigE recognition motifs are created/disrupted at a lower rate than expected by chance.

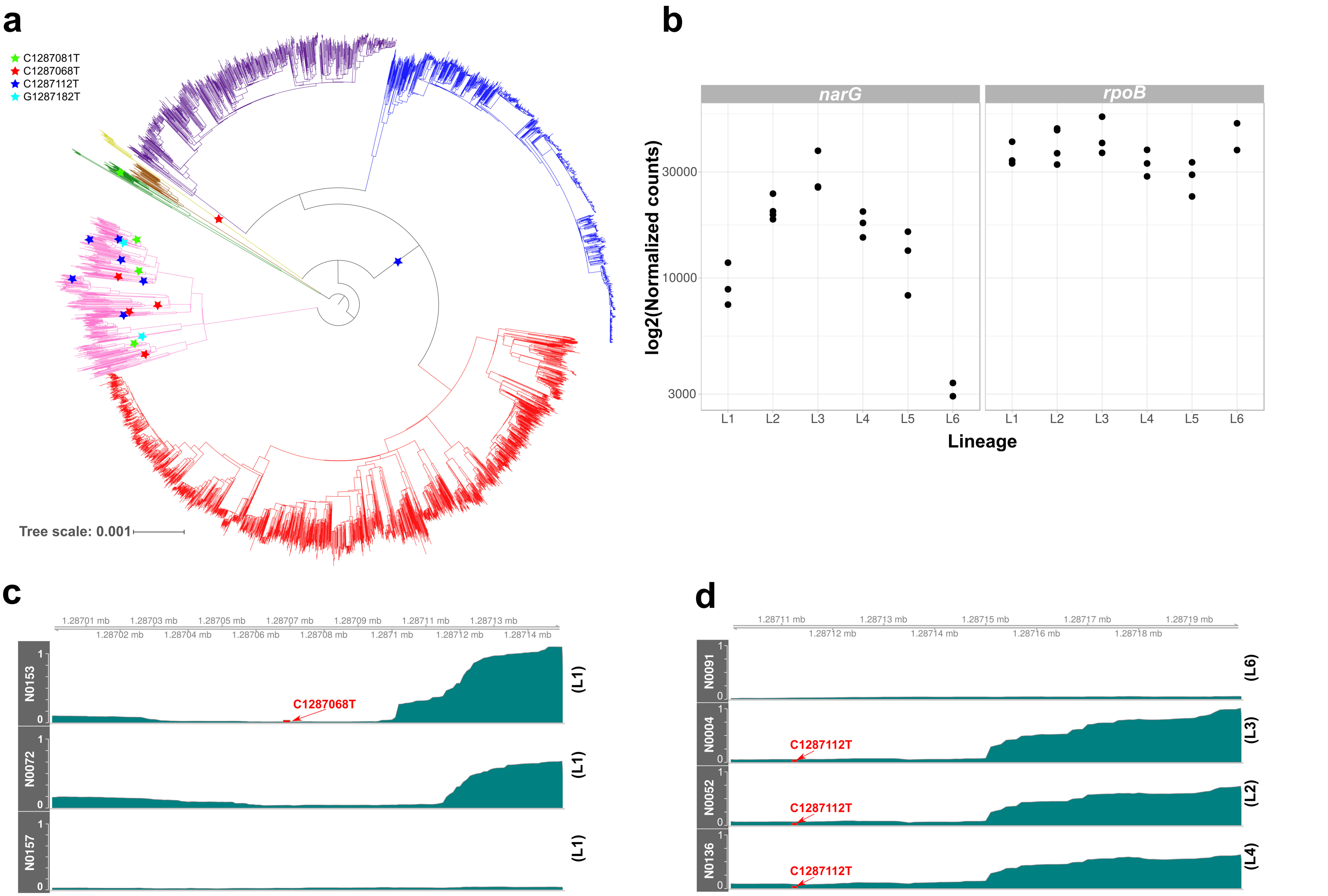

Supplementary Figure 4. a,The *narG* promoter mutations that create new Pribnow boxes are highly homoplastic. The phylogeny plotted was constructed by using the maximum-likelihood method, with the 4,595 strains dataset. **b**, Comparative between the expression values (y-axis, log of the normalized counts) of the *narG* gene and the housekeeping gene *rpoB* for the different lineages (x-axis). **c**, The new Pribnow box present in the N0153 strains upregulates the expression of *narG* in contrast to the other L1 strains. **d**, The new Pribnow box in the common branch of the modern lineages upregulates the transcription of *narG* in contrast to L6 strains.

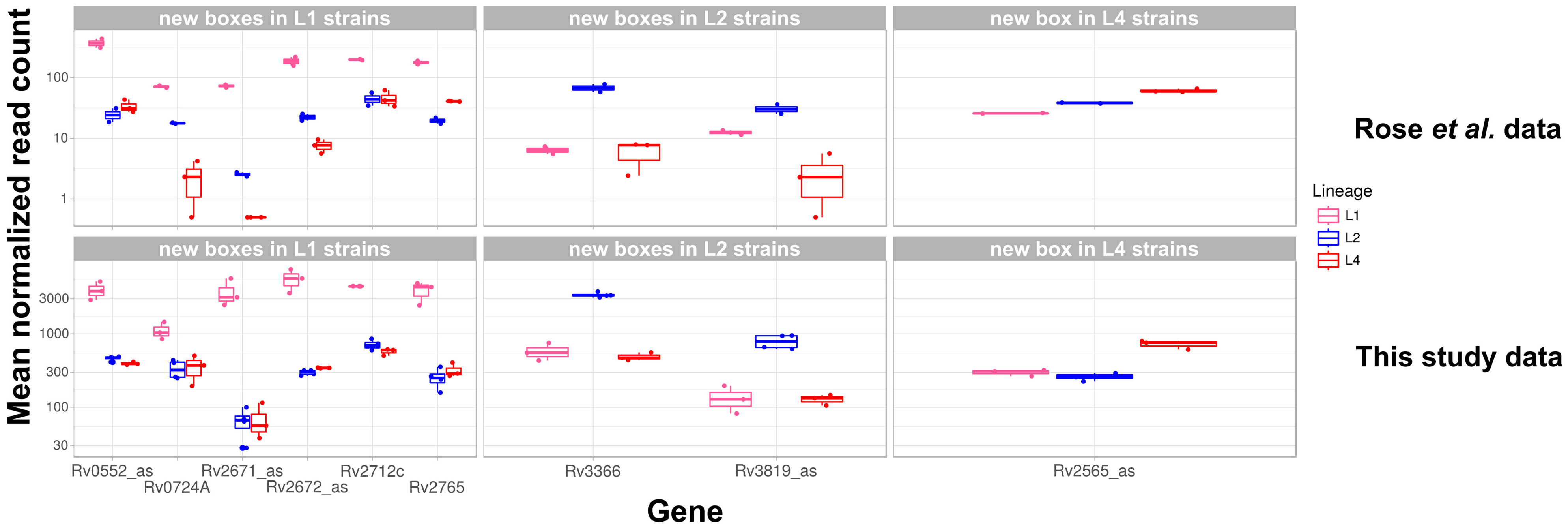

Supplementary Figure 5. Effect of the new Pribnow boxes over gene expression tested in independent datasets. New Pribnow boxes generated by point mutations increase the expression of nearby genes in the strains in which the new box appears. The observed effect is independent of the RNAseq dataset used. When the new Pribnow box appears in the noncoding strand, the increase in expression is observed in the antisense RNA (denoted in the x-axis as '_as'). From the Rose *et al.* dataset[9], strains N0145 (L2), N0153 (L1) and H37Rv (L4) were used.

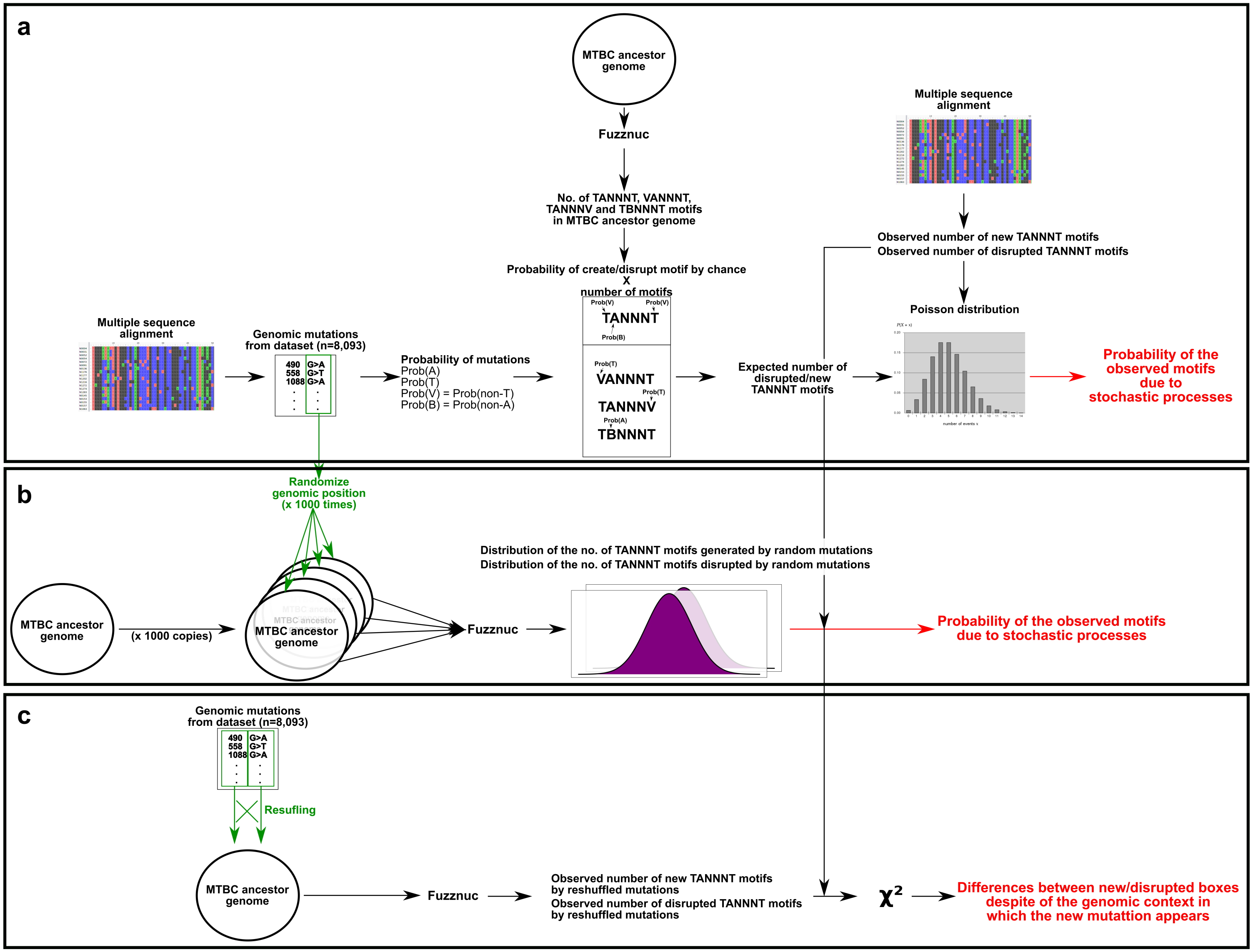

Supplementary Figure 6. Statistical analysis performed to evaluate if non-random process are influencing the observed number of new and disrupted Pribnow boxes. a, Probability of the observed creation/disruption of Pribnow boxes, given the probability of each genomic position to accumulate variants. **b**, Permutation test to assess the number of new/disrupted boxes expected by random mutations impacting the MTBC genome. **c**, Chi-squared distribution of expected vs observed ratio of new/disrupted boxes, when the mutations in the dataset are reshuffled.

Supplementary References

1. Castell, A., Johansson, P., Unge, T., Jones, T. A. & Bäckbro, K. Rv0216, a conserved hypothetical protein from *Mycobacterium tuberculosis* that is essential for bacterial survival during infection, has a double hotdog fold. *Protein Sci.* **14**, 1850–1862 (2005).
2. Takayama, K., Wang, C. & Besra, G. S. Pathway to synthesis and processing of mycolic acids in *Mycobacterium tuberculosis*. *Clin. Microbiol. Rev.* **18**, 81–101 (2005).
3. Gupta, A., Venkataraman, B., Vasudevan, M. & Bankar, K. G. Co-expression network analysis of toxin-antitoxin loci in Mycobacterium tuberculosis reveals key modulators of cellular stress. *Sci. Rep.* **7**, 5868 (2017).
4. Vandal, O. H., Nathan, C. F. & Ehrt, S. Acid resistance in *Mycobacterium tuberculosis*. *J. Bacteriol.* **191**, 4714–4721 (2009).
5. Darwin, K. H. *Mycobacterium tuberculosis* and copper: A newly appreciated defense against an old foe? *J. Biol. Chem.* **290**, 18962–18966 (2015).
6. Keating, L. A. *et al.* The pyruvate requirement of some members of the *Mycobacterium tuberculosis* complex is due to an inactive pyruvate kinase: implications for in vivo growth. *Mol. Microbiol.* **56**, 163–174 (2005).
7. Akhtar, S., Khan, A., Sohaskey, C. D., Jagannath, C. & Sarkar, D. Nitrite reductase NirBD is induced and plays an important role during in vitro dormancy of *Mycobacterium tuberculosis*. *J. Bacteriol.* **195**, 4592–4599 (2013).
8. Singh, A. *et al.* Requirement of the *mymA* operon for appropriate cell wall ultrastructure and persistence of *Mycobacterium tuberculosis* in the spleens of guinea pigs. *J. Bacteriol.* **187**, 4173–4186 (2005).

9. Rose, G. *et al.* Mapping of genotype–phenotype diversity among clinical isolates of *Mycobacterium tuberculosis* by sequence-based transcriptional profiling. *Genome Biol. Evol.* **5**, 1849 (2013).
10. Yruela, I., Contreras-Moreira, B., Magalhães, C., Osório, N. S. & Gonzalo-Asensio, J. *Mycobacterium tuberculosis* complex exhibits lineage-specific variations affecting protein ductility and epitope recognition. *Genome Biol. Evol.* **8**, 3751–3764 (2016).
11. Dawes, S. S. *et al.* Ribonucleotide reduction in *Mycobacterium tuberculosis*: function and expression of genes encoding class Ib and class II ribonucleotide reductases. *Infect. Immun.* **71**, 6124–6131 (2003).
12. Lew, J. M., Kapopoulou, A., Jones, L. M. & Cole, S. T. TubercuList – 10 years after. *Kekkaku* **91**, 1–7 (2011).
13. Marjanovic, O., Miyata, T., Goodridge, A., Kendall, L. V. & Riley, L. W. Mce2 operon mutant strain of *Mycobacterium tuberculosis* is attenuated in C57BL/6 mice. *Tuberculosis* **90**, 50–56 (2010).
14. Rodionov, D. A. & Gelfand, M. S. Identification of a bacterial regulatory system for ribonucleotide reductases by phylogenetic profiling. *Trends Genet.* **21**, 385–389 (2005).
15. Young, D. B., Comas, I. & de Carvalho, L. P. S. Phylogenetic analysis of vitamin B12-related metabolism in *Mycobacterium tuberculosis*. *Frontiers in Molecular Biosciences* **2**, (2015).
16. Iona, E. *et al. Mycobacterium tuberculosis* gene expression at different stages of hypoxia-induced dormancy and upon resuscitation. *J. Microbiol.* **54**, 565–572 (2016).
17. Chiner-Oms, Á., González-Candelas, F. & Comas, I. Gene expression models based on a reference laboratory strain are poor predictors of *Mycobacterium tuberculosis* complex transcriptional diversity. *Sci. Rep.* **8**, 3813 (2018).

18. Chiner-Oms, Á. *et al.* Genomic determinants of speciation and spread of the *Mycobacterium tuberculosis* complex. *Science Advances* In press

19. Coscolla, M. & Gagneux, S. Consequences of genomic diversity in Mycobacterium tuberculosis. *Semin. Immunol.* **26**, 431–444 (2014).
