# Supplementary material for: Genome-wide mutational biases fuel transcriptional diversity in the Mycobacterium tuberculosis complex
Source: Nat Commun. 2019 Sep 5;10:3994. doi: 10.1038/s41467-019-11948-6 (PMC6728331; doi:10.1038/s41467-019-11948-6)
Supplement: Supplementary file 3 — Reporting Summary [file 41467_2019_11948_MOESM3_ESM.pdf]

nature research

Corresponding author(s): Iñaki Comas

Last updated by author(s): Jul 4, 2019

Reporting Summary

Nature Research wishes to improve the reproducibility of the work that we publish. This form provides structure for consistency and transparency in reporting. For further information on Nature Research policies, see Authors & Referees and the Editorial Policy Checklist.

Statistics

For all statistical analyses, confirm that the following items are present in the figure legend, table legend, main text, or Methods section.

n/a	Confirmed	
☐	☒	The exact sample size (*n*) for each experimental group/condition, given as a discrete number and unit of measurement
☐	☒	A statement on whether measurements were taken from distinct samples or whether the same sample was measured repeatedly
☐	☒	The statistical test(s) used AND whether they are one- or two-sided<br>*Only common tests should be described solely by name; describe more complex techniques in the Methods section.*
☒	☐	A description of all covariates tested
☐	☒	A description of any assumptions or corrections, such as tests of normality and adjustment for multiple comparisons
☐	☒	A full description of the statistical parameters including central tendency (e.g. means) or other basic estimates (e.g. regression coefficient) AND variation (e.g. standard deviation) or associated estimates of uncertainty (e.g. confidence intervals)
☐	☒	For null hypothesis testing, the test statistic (e.g. *F*, *t*, *r*) with confidence intervals, effect sizes, degrees of freedom and *P* value noted<br>*Give P values as exact values whenever suitable.*
☒	☐	For Bayesian analysis, information on the choice of priors and Markov chain Monte Carlo settings
☒	☐	For hierarchical and complex designs, identification of the appropriate level for tests and full reporting of outcomes
☒	☐	Estimates of effect sizes (e.g. Cohen's *d*, Pearson's *r*), indicating how they were calculated

Our web collection on statistics for biologists contains articles on many of the points above.

Software and code

Policy information about availability of computer code

Data collection	In the genomic analysis pipelines the main open source programs used were the SMRT Analysis Pipeline (SMRT® Analysis Server v2.3.0), fastp (0.12.5), BWA (0.7.12-r1039), samtools (1.3.1), picard-tools (2.18.0), VarScan (2.3.7), GATK (3.8-1-0-gf15c1c3ef), BedTools (2.26.0), emboss (6.6.0.0).
Data analysis	Statistical analyses were performed by using the R programming language (version 3.4.4) to manage and analyze the data, as well as to generate the plots. Details of the main R packages used can be found in the Methods section of the manuscript.

For manuscripts utilizing custom algorithms or software that are central to the research but not yet described in published literature, software must be made available to editors/reviewers. We strongly encourage code deposition in a community repository (e.g. GitHub). See the Nature Research guidelines for submitting code & software for further information.

Data

Policy information about availability of data

All manuscripts must include a data availability statement. This statement should provide the following information, where applicable:

- Accession codes, unique identifiers, or web links for publicly available datasets
- A list of figures that have associated raw data
- A description of any restrictions on data availability

All the new data generated for the present study was submitted to ENA under accession numbers PRJEB8783 (PacBio data) and PRJEB9763 (transcriptomic data). Whole-genome sequencing of N0153, N117 and N1063 was deposited under accession PRJEB31443, while the sequence data from the rest of the samples comes from another publication and can be found under accession number PRJEB27802. The source data underlying Figs 1a, 1b, 2b, 3a, 3b, 4a, 5b, 6a and 6b are provided as a Source Data file. All other relevant data is available upon request.

Field-specific reporting

Please select the one below that is the best fit for your research. If you are not sure, read the appropriate sections before making your selection.

☒ Life sciences ☐ Behavioural & social sciences ☐ Ecological, evolutionary & environmental sciences

For a reference copy of the document with all sections, see nature.com/documents/nr-reporting-summary-flat.pdf

Life sciences study design

All studies must disclose on these points even when the disclosure is negative.

Sample size	Samples were selected according to their position in the MTBC phylogeny, with the objective of maximizing the genetic variability found in the complex (see Supplementary Figure 1).
Data exclusions	As stated in the main text, only sample N1177 were excluded from the transcriptomic analyses as it carries a mutation in the rpoB gene that alters its transcriptomic profile.
Replication	As specified in the Results and Methods section, two replicates per sample were generated. The transcriptomic analysis of each replicate shows a high agreement.
Randomization	Not applicable
Blinding	Not applicable

Reporting for specific materials, systems and methods

We require information from authors about some types of materials, experimental systems and methods used in many studies. Here, indicate whether each material, system or method listed is relevant to your study. If you are not sure if a list item applies to your research, read the appropriate section before selecting a response.

Materials & experimental systems

n/a	Involved in the study	
☒	☐	Antibodies
☒	☐	Eukaryotic cell lines
☒	☐	Palaeontology
☒	☐	Animals and other organisms
☒	☐	Human research participants
☒	☐	Clinical data

Methods

n/a	Involved in the study	
☒	☐	ChIP-seq
☒	☐	Flow cytometry
☒	☐	MRI-based neuroimaging
