# Supplementary material for: Genome-wide mutational biases fuel transcriptional diversity in the Mycobacterium tuberculosis complex
Source: Nat Commun. 2019 Sep 5;10:3994. doi: 10.1038/s41467-019-11948-6 (PMC6728331; doi:10.1038/s41467-019-11948-6)
Supplement: Supplementary file 4 — Description of Additional Supplementary Files [file 41467_2019_11948_MOESM4_ESM.pdf]

Description of Additional Supplementary Files

File Name:Supplementary Data 1
Description: Mutations found in the pyruvate kinase gene *pykA* (Rv1617) in the cultured strains

File Name:Supplementary Data 2
Description: Accession numbers for the samples

File Name:Supplementary Data 3
Description: Number of Phylogenetically Aware Differentially Expressed genes and the branch length for each of the main clades in the MTBC phylogeny. The phylogeny was calculated by using the Maximum-Likelihood algorithm, with 1,000 iteration bootstrapping.

File Name:Supplementary Data 4
Description: Phylogenetically Aware Differentially Expressed genes for the comparison between the main phylogenetic groups. BaseMean, log2FoldChange and padj (BH) values were calculated with the DEseq2 package. The branch assignation was obtained while comparing the branch again the rest of the MTBC (see Methods).

File Name:Supplementary Data 5
Description: Gene Ontology enrichment analysis for the up and down-regulated genes for each branch of the PDGE analysis.

File Name:Supplementary Data 6
Description: SNPs that either create or disrupt Pribnow boxes, and their potential effect over the expression of specific genes.

File Name:Supplementary Data 7
Description: Methylation profile of the 19 MTBC samples. Summary of the methylation report derived from the PacBio sequencing for each of the three main methyltransferases. For each motif, the methyltransferase involved in its methylation and the number of occurrences identified in the genome are reported in the header. In almost all samples, all the motifs identified are methylated. In those samples having less than 80% of the motifs methylated, non-synonymous variants were identified in the methyltransferase coding genes.

File Name:Supplementary Data 8
Description: Non-synonymous SNPs affecting the three main methyltransferases found in an MTBC dataset (n=4,595) representative of the global MTBC diversity.

File Name:Supplementary Data 9
Description: Differential expression analysis performed with DEseq2, for N1283-Δ*hsdM* and N1283 strains.

File Name:Supplementary Data 10
Description: Genes identified as carrying deletions relative to the H37Rv reference strain. Deletions were identified by mapping the sequenced long-reads against the H37Rv genome.

File Name:Supplementary Data 11

Description: Genes enriched for repeated regions, not taken into account for the RNA-seq and variant analyses

File Name:Supplementary Data 12
Description: PCR primers used for the *hsdM* deletion confirmation
